# Heat Shock Proteins and Ferroptosis

**DOI:** 10.3389/fcell.2022.864635

**Published:** 2022-04-11

**Authors:** Ying Liu, Lin Zhou, Yunfei Xu, Kexin Li, Yao Zhao, Haoduo Qiao, Qing Xu, Jie Zhao

**Affiliations:** ^1^ Department of Neurosurgery, Xiangya Hospital, Central South University, Changsha, China; ^2^ Department of Pathophysiology, Xiangya School of Medicine, Central South University, Changsha, China; ^3^ Sepsis Translational Medicine Key Lab of Hunan Province, Changsha, China; ^4^ China-Africa Research Center of Infectious Diseases, Central South University, Changsha, China

**Keywords:** ferroptosis, heat shock proteins, ubiquitin, molecular chaperone, GPx4

## Abstract

Ferroptosis is a new form of regulatory cell death named by Dixon in 2012, which is characterized by the accumulation of lipid peroxides and iron ions. Molecular chaperones are a class of evolutionarily conserved proteins in the cytoplasm. They recognize and bind incompletely folded or assembled proteins to help them fold, transport or prevent their aggregation, but they themselves do not participate in the formation of final products. As the largest number of molecular chaperones, heat shock proteins can be divided into five families: HSP110 (HSPH), HSP90 (HSPC), HSP70 (HSPA), HSP40 (DNAJ) and small heat shock proteins (HSPB). Different heat shock proteins play different roles in promoting or inhibiting ferroptosis in different diseases. It is known that ferroptosis is participated in tumors, nervous system diseases, renal injury and ischemia-reperfusion injury. However, there are few reviews about the relationship of heat shock proteins and ferroptosis. In this study, we systematically summarize the roles of heat shock proteins in the occurrence of ferroptosis, and predict the possible mechanisms of different families of heat shock proteins in the development of ferroptosis.

## Introduction

Ferroptosis is an iron-dependent regulatory cell death mode, which is different from apoptosis, necrosis and pyrodeath in morphology, biochemistry and other aspects. Studies have found that ferroptosis is not only related to tumorigenesis, but also involved in the occurrence of a variety of nervous system diseases. What’s more, it also plays an important role in many diseases such as ischemia-reperfusion injury.

The ability of cells to respond to external stress is important for homeostasis, and stress of all kinds is particularly harmful for proteins that need to fold to function (Morimoto and Cuervo, 2014). Folded proteins have very little thermodynamic stability and are susceptible to environmental changes even when cell conditions change slightly (Kim Y. E. et al., 2013). To do this, a complex network of networks formed inside the cell to cope with these stressful conditions, that is, a quality control system for proteins (Thirumalai et al., 2020). The system regulates the entire process from initial synthesis of proteins on ribosomes to final degradation of proteins. As main members of this protein quality control system, molecular chaperones play an indispensable role in maintaining protein stability. Among them, heat shock proteins (HSP) are the most abundant molecular chaperone proteins. In addition to normal expression under physiological conditions, HSPs expression increases when cells are exposed to stress conditions, such as high temperature or hypoxia (Rylander et al., 2005). According to the order of molecular weight, HSPs were classified by predecessors, including HSP110, HSP90, HSP70, HSP40 and small heat shock protein (sHSP) (Kampinga et al., 2009). What is more, there are also HSP related proteins, such as co-chaperone, TCP-1 ring complex (TriC), protein disulfide isomerases (PDIs) and cis-trans proline isomerase, cadherin/calreticulin, etc., (Hageman and Kampinga, 2009; Kampinga et al., 2009).

In recent years, more and more studies have shown that HSPs participate in the pathophysiological process of ferroptosis. They play different roles in the process of ferroptosis in occurrence, development and regulation. Therefore, this paper makes a systematic review on the regulation of HSPs on ferroptosis, in which we focus on the important role of different HSPs in the process of ferroptosis, which may provide innovative ideas for further research on ferroptosis.

## Ferroptosis

Regulated cell death (RCD) is an important process that maintains the metabolism and homeostasis of multicellular organisms, including apoptosis, necrosis, pyroptosis, ferroptosis and other forms of RCD (Galluzzi et al., 2018; Tang et al., 2019). Among them, ferroptosis has been revealed to be involved in more and more diseases in recent years, such as neurodegenerative diseases (Ayton et al., 2015), cancer (Chen X. et al., 2021), ischemia-reperfusion injury (IRI) (Swaminathan, 2018), intestinal diseases (Xu S. et al., 2021) and so on. Unlike apoptosis (cell atrophy, chromatin concentration, cytoskeleton disintegration and the formation of apoptotic bodies), necrosis (cell membrane rupture, organelle swelling) and pyroptosis (cell swelling, nuclear shrinkage, cell membrane rupture and content leakage), the biochemical aspects of ferroptosis are characterized by the accumulation of intracellular iron and lipid reactive oxygen. As major sites of iron utilization and master regulators of oxidative metabolism, mitochondria are the main source of reactive oxygen species (ROS) and, thus, the severity of damage to mitochondrial morphology, bioenergetics and metabolism is closely related to ferroptosis (Battaglia et al., 2020). Morphologically, when ferroptosis occurs in cells, the volume of mitochondria becomes smaller, accompanied by the increase of mitochondrial membrane density, the decrease or disappearance of cristae and the rupture of mitochondrial outer membrane, which may be caused by the dysfunction of voltage-dependent anion channels (VDCAs) and the change of mitochondrial membrane fluidity caused by lipid peroxidation products (DeHart et al., 2018). Although ferroptosis is widely involved in the pathological process of many different diseases, there is still no specific molecular detection index for it. The existence of ferroptosis can only detected by the level of lipid peroxidation and the inhibition of ferroptosis inhibitors or iron chelators on cell death.

In recent years, the complex and unknown regulatory mechanism network of ferroptosis is slowly being revealed, including cysteine/glutamate antiporter system (system Xc^−^), glutathione peroxidase (GPX), mitochondrial membrane voltage dependent anion channel, iron metabolism, ferroptosis suppressor protein 1(FSP1), GTP cyclohydrolase 1 (GCH1), p53, HO-1, p62-Keap1-Nrf2, ATG5-ATG7-NCOA4 and other related signal pathways. This paper will focus on the mechanism of ferroptosis from five aspects: iron metabolism, GSH/GPX4 antioxidant pathway, FSP1/CoQ10 antioxidant pathway, GCH1/BH4 antioxidant pathway, lipid peroxidation and the production of reactive oxygen species.

### Iron Metabolism

Iron is one of the most basic life-sustaining elements and is involved in many physiological processes, such as oxygen transport, cell respiration, DNA synthesis, myelination and neurotransmitter biosynthesis. Hemoglobin in the blood contains iron, which carried by red blood cells and carries oxygen through the blood circulation to different tissues to maintain the body’s blood oxygen supply (Martínková et al., 2013). Most of the iron in the human body is mainly stored in hemoglobin and myosin, the rest combined with iron storage proteins, such as ferritin, transferrin, cytochrome, etc. There is no doubt that this is necessary for normal cell and tissue physiology.

Under normal conditions, iron exists in the form of Fe^2+^ and Fe^3+^. However, during the intracellular iron cycle, iron binds to transferrin (TF) in the form of Fe^3+^, is absorbed into cells through transferrin receptor 1 (TFR1) and stored in endosomes. In the endosomes, Fe^3+^ is transformed into Fe^2+^ by six-transmembrane epithelial antigen of prostate 3(STEAP3) (Yan and Zhang, 2019). Then Fe^2+^ is transported from endosomes to cytosol via divalent metal transporter 1 (DMT1) (Xie et al., 2016). After that, some Fe^2+^ is stored in ferritin heavy chain 1(FTH1) and ferritin light chain (FLC) of iron storage protein complex, while others are transported out of cells through ferroportin1(FPN1). FPN1 is the only known protein that controls iron output in mammalian cells, and plays a critical role in the transport of Fe^2+^ and maintenance of intracellular iron balance (Trujillo-Alonso et al., 2019). However, based on this regulated mechanism of precise intracellular input-transport-storage or exocytosis, any process of iron uptake, transportation, storage or utilization will cause abnormal metabolism of intracellular iron ions, resulting in abnormalities in the Fe^2+^ metabolic chain, and then may lead to Fenton reaction to generate ROS (Xu et al., 2020). Then, ROS subsequently modifies and interferes with proteins, lipids and DNA, and finally induces the termination of cell life (Singh et al., 2014; Bogdan et al., 2016). It is worth noting that the release of mitochondrial ROS caused by mitochondrial damage also aggravates this process and promotes ferroptosis (Wu et al., 2021).

### Glutathione/Glutathione Peroxidase 4 Antioxidant Pathway

Cells transport substances to the cells for digestion, absorption and uptake of nutrients to maintain biochemical reactions. Nutrients such as sugar, fat and amino acids cannot directly enter cells, but they must transported through specific transporters. System Xc^−^, a cysteine/glutamate reverse transporter widely distributed in phospholipid bilayer, is one of these transporters. System Xc^−^ is a disulfide heterodimer composed of light chain subunit (SLC7A11) and heavy chain subunit (SLC3A2). It plays an important role in the upstream events of ferroptosis and is responsible for 1:1 input of cystine and excretion of glutamate (Dixon et al., 2012). Cystine transported into the cell and reduced to cysteine. In addition to relying on System Xc^−^, cysteine can also enter cells through sulfur-transfer pathway. Cysteine entering cells through these two pathways and becomes the raw material for the synthesis of glutathione (GSH) and participates in the synthesis of GSH (Xu W. et al., 2021). GSH is the core substance of amino acid metabolism in the process of ferroptosis (Friedmann Angeli et al., 2014; Yang et al., 2014), which is an important antioxidant to protect cells from oxidative damage and is also the substrate of GPX4 lipid repair function. In the process of ferroptosis, GPX4 as the central regulator is an important enzyme that scavenge anaerobic free radicals from lipids (Yang et al., 2014). Once activated, GPX4 catalyzes toxic lipid hydroperoxides (L-OOH) into non-toxic lipid alcohols (L-OH) (Ursini et al., 1982). Under this catalysis, GPX4 uses two molecules of GSH as substrate to produce one molecule of oxidized glutathione (GSSG). GSSG reduced to GSH by GSH reductase in a NADPH dependent manner. Overexpression of GPX4 can inhibit ROS production and lipid peroxidation (Koppula et al., 2018), while the decrease of GPX4 activity or expression will lead to the accumulation of intracellular lipid peroxide, resulting in ferroptosis (Kim et al., 2021). Thus, the expression of GPX4 may represent the process of ferroptosis to some extent. It is worth mentioning that some studies have shown that knockout of GPX4 can also cause cell death and tissue damage by means of necrosis (Canli et al., 2016), pyroptosis (Kang et al., 2018) or apoptosis (Ran et al., 2007). Therefore, it has important pathological significance to continue to explore the role and mechanism of GPX4.

### Ferroptosis Suppressor Protein 1/CoQ10 Antioxidant Pathway

Initially, the GSH/GPX4 pathway was thought to be the only ferroptosis inhibiting pathway, but later it was found that there is another parallel inhibitory pathway: in the absence of GPX4, ferroptosis suppressor protein 1 (FSP1) adequately counteracts lethal lipid peroxidation and ferroptosis (Bersuker et al., 2019; Doll et al., 2019). FSP1 is a member of the type II NADH: quinone oxidoreductase (NDH-2) family. Thus, its intrinsic role is to reduce CoQ10 using NADH (Elguindy and Nakamaru-Ogiso, 2015). During this resistance to ferroptosis, FSP1 terminates lipid autoxidation by reducing ubiquinone to ubiquinol, which in turn can directly reduce lipid free radicals. In addition, it prevents lipid peroxidation and ferroptosis by regenerating oxidized α-tocopherol radicals, the most powerful natural chain-breaking antioxidants in lipids, into non-radical forms.

### GCH1/BH4 Antioxidant Pathway

As research on ferroptosis progresses more rapidly, another highly effective endogenous ferroptosis inhibitor, GTP Cyclohydrolase 1 (GCH1), was reported by Kraft in 2020 (Kraft et al., 2020). GCH1 is the rate-limiting enzyme for the synthesis of the antioxidant tetrahydrobiopterin (BH4). Like FSP1/CoQ10 axis, GCH1/BH4 pathway also blocks ferroptosis in a GPX4-independent form. In this process, BH4 needs to regenerated by reducing BH2 by dihydrofolate reductase (DHFR). What’s more, BH4 converts phenylalanine to tyrosine, which in turn is converted to 4-OH-benzoate (a precursor of CoQ10) to promote the synthesis of CoQ10, thereby inhibiting ferroptosis.

### Production of Reactive Oxygen Species and Lipid Peroxidation

ROS is a by-product of cellular oxygen metabolism, which is responsible for maintaining the stability of the body and participating in the signal transduction of cell normal metabolism. ROS include peroxide (H_2_O_2_、ROOH) superoxide (O_2_^−^) singlet oxygen (^1^O_2_) and free radicals (HO^−^, HO_2_^−^, R^−^, NO^−^and NO_2_^−^). Under pathological conditions, excessive ROS can destroy all types of cellular components, such as nucleic acids, proteins and lipids, resulting in cell death (Imlay, 2013). Most ferroptosis related ROS come from Fenton reaction and Haber Weiss reaction (Shen et al., 2018), then ROS interacts with polyunsaturated fatty acids (PUFAs) on lipid membrane to form lipid ROS. When a large amount of lipid ROS accumulates in cells, it will cause ferroptosis (Florean et al., 2019).

Lipid peroxidation refers to the lipid oxidative degradation reaction in which lipids lose hydrogen atoms under the action of free radicals or lipid peroxidase, resulting in the oxidation, fracture and shortening of lipid carbon chain, and produce cytotoxic substances resulting in cell damage (Ayala et al., 2014). More and more studies have shown that lipid peroxide is a key mediator of many pathological states, including inflammation (Kang and Yang, 2020), cancer (Rodríguez-García et al., 2020) and neurodegenerative diseases (Zhao et al., 2021). Among them, lipid peroxides have toxic effects on cells mainly through two mechanisms: at the molecular level, lipid peroxides are further decomposed into active substances, which consume nucleic acids and proteins, leading to ferroptosis; at the structural level, lipid peroxidation leads to thinning and increased bending of the biofilm, which ultimately leads to membrane instability and micellar formation (Gaschler and Stockwell, 2017).

Before lipid peroxidation, activated long-chain acyl CoA synthase 4 (ACSl4) induced esterification of free PUFAs and incorporated into membrane phospholipids with the assistance of lysophosphatidylcholine acyltransferase 3 (LPCAT3). Therefore, the upregulation of ACSL4 generally regarded as a sign of ferroptosis. In this process, the level and location of intracellular PUFAs determine the degree of lipid peroxidation (Liu et al., 2021). It is worth noting that free PUFAs, such as arachidonic acid (AA) and adrenaline (AdA), must be esterified into phospholipids (PLs) for peroxidation (Sato et al., 1999). Subsequently, lipoxygenases (LOXs), such as cyclooxygenases (COXs) and cytochrome P450 enzymes (CYPs), catalyze PL-PUFAs to form lipid hydroperoxides (Doll and Conrad, 2017; Lei et al., 2019). As mentioned above, GPX4 reduces PL-PUFAs lipid peroxides (L-OOH) to lipid alcohols (L-OH). Excess iron ions in the cytoplasm trigger free radical induced lipid hydroperoxides that damage cells. In addition, these free radicals can transfer protons, leading to a new wave of lipid peroxidation with positive feedback.

## Heat Shock Proteins and Ferroptosis

Molecular chaperones are a class of evolutionarily conserved proteins in the cytoplasm. They recognize and bind incompletely folded or assembled proteins to help them fold, transport or prevent their aggregation. However, molecular chaperones themselves do not participate in the formation of final products (Hoter et al., 2018). In cell homeostasis, molecular chaperones regulate the activity and interaction of mature proteins by assisting in the proper folding and assembly of newborn polypeptide chains, guide damaged or short-lived proteins into the degradation pathway. All these chaperones work together to maintain the mass and weight balance of protein and avoid misfolding and/or aggregation of client proteins.

Based on the naming method specified by HUGO Gene Nomenclature Committee, the HSP family divided into five subfamilies according to molecular weight: HSP110 (HSPH), HSP90 (HSPC), HSP70 (HSPA), HSP40 (DNAJ) and small HSP (HSPB). Although, the latest nomenclature contains heat shock 70 kDa proteins, DNAJ (HSP40) heat shock proteins, small heat shock proteins, heat shock 90 kDa proteins, and chaperonins.

Based on different molecular weights, different types of HSPs also have great differences in configuration. For example, the macromolecule HSP104 forms a hexameric ring and promotes unfolding through a ratchet mechanism. HSP90 forms a multi domain V-shaped structure, and its scissors like movement helps to refine the receptor protein. HSP70 and small HSP use modular “clamps” to protect the extended hydrophobic structure in the target protein. HSP60 adopts barrel “Anfinsen” cage structure for isolation and folding of target protein (Bascos and Landry, 2019).

Ferroptosis is an oxidative stress-dependent regulation of cell death related to iron accumulation and lipid peroxidation. Since it named in 2012, there are still many unexplored mechanisms (Dixon et al., 2012). Members of the HSP family play an important role in the occurrence and development of ferroptosis. However, there are few relevant reviews on the interaction between molecular chaperones and ferroptosis. In this paper, the existing research results on molecular chaperones in ferroptosis reviewed to provide clues for further revealing the unknown mechanism of ferroptosis and to clarify the potential role of molecular chaperones in ferroptosis.

### HSP90 (HSPC) and Ferroptosis

HSP90 family is one of the most widely studied molecular chaperones of HSP family. It is also one of the most abundant proteins in cells, accounting for 1–2% of cellular proteins and 4–6% in stressed cells (Picard, 2002; Taipale et al., 2010).HSP90 family include cytoplasmic HSP90*α* and HSP90*β*, endoplasmic reticulum glucose regulatory protein 94 (GRP94) and mitochondrial TNF receptor associated protein 1 (TRAP1) (DeZwaan and Freeman, 2010). HSP90 have conserved domains in the process of evolution, including an amino terminal domain (NTD) and a carboxyl terminal domain (CTD) (Csermely et al., 1998). The evolutionary conservation and expression universality reflect the basic and necessary role of HSP90 in cell physiology.

As ATPase, ATP binding and hydrolysis are the key characteristics of HSP90’s activity on client proteins. In addition to the hydrolysis of ATP, the function of HSP90 also regulated by its interacting co-chaperones (Sreedhar et al., 2004; Zhao et al., 2005; Prodromou, 2016). In addition, the role of HSP90 is also essential in genomic DNA. In mammalian cells, HSP90 enhances the enzymatic activity of histone methyltransferase SMYD3, which is involved in RNA polymerase II-mediated transcriptional regulation (Hamamoto et al., 2004). Moreover, *Drosophila* HSP90 also participates in the interaction with chromatin modifying enzyme trithorax protein, so as to participate in gene regulation (Tariq et al., 2009).

Compared with other HSPs, HSP90 has received more attention as a promising anticancer drug target because of its clear importance in regulating the stability and activity of many proteins involved in human cancer. It is now clear that siRNA interference with HSP90 can protect mouse nerve cells from the toxic effect of ferroptosis (Wu et al., 2019). Besides, necrotic apoptosis and ferroptosis are two different ways of necrotic cell death, there is no common regulatory mechanism. Studies have shown that HSP90 plays a complex role in necrotic apoptosis by binding and regulating the activity of RIPK1, RIPK3 or MLKL in a strictly cell environmental-dependent manner (Li et al., 2015; Jacobsen et al., 2016). However, recent studies have found that chaperone mediated autophagy (CMA) can be used as a common regulatory point to regulate necrotic apoptosis and ferroptosis (Wu et al., 2019). CMA degrades its substrate GPX4 by interacting with Lamp-2a and GPX4-HSC70-HSP90 trimers located in lysosomes. Inhibition of CMA can stabilize GPX4 and reduce ferroptosis. Furthermore, in IRI or folate-induced acute kidney injury (AKI) models, HSC70-HSP90-GPX4 interacts with legumain in the kidney to promote chaperone mediated degradation of GPX4, thereby promoting renal tubular cell ferroptosis in AKI (Chen C. A. et al., 2021).

Therefore, based on the existing scientific research results, HSP90 family may act on GPX4, inhibit the antioxidant capacity of GPX4 by inhibiting its activity (Zhou et al., 2021), then participate in the regulation of ferroptosis through GSH/GPX4 pathway and inhibite lipid peroxidation, therefore influencing ferroptosis.

### HSP70 (HSPA) and Ferroptosis

The molecular chaperones of HSP70 family exist in different forms, including cytoplasmic HSP73 (also named HSC70), cytoplasmic inducible HSP72, mitochondrial HSP75/lethal protein mortalin and endoplasmic reticulum HSP78/BIP. Other variants include HSP72 and HSP70i (Kim et al., 2020).

The neuroprotective properties of HSP70 have been widely studied. In the ischemic stroke model, HSP70 plays an endogenous protective mechanism in the penumbra of the hippocampus (States et al., 1996; Weinstein et al., 2004). And HSP70 can protect cells from apoptosis, necrosis and other types of cell death (Frebel and Wiese, 2006). It is reported that HSP70 seems to improve nerve injury by blocking cell death and immune response. Besides, HSP70 is also involved in the regulation of inflammatory pathways (Kim J. Y. et al., 2013). Therefore, more and more drugs take HSP70 as a potential therapeutic target for nervous system diseases.

HSPA5, also known as GRP78 or Bip, is an important member of HSP70 family. As a receptor of endoplasmic reticulum stress, HSPA5 is responsible for regulating the folding and transport of unfolded proteins in endoplasmic reticulum, so as to maintain the homeostasis. In human pancreatic ductal adenocarcinoma cells (PDAC), HSPA5 negatively regulates ferroptosis of PDAC cells through HSPA5-GPX4 signaling pathway, and mediates resistance to ferroptosis (Zhu et al., 2017). Similarly, dihydroartemisinin (DHA) can induce ferroptosis in glioma cells. This is because DHA causes endoplasmic reticulum stress in glioma cells, which up regulates the expression of activated transcription factor 4 (ATF4) and induces the expression of HSPA5 by activating protein kinase R-like endoplasmic reticulum kinase (PERK). The upregulation of HSPA5 increases the expression and activity of GPX4, GPX4 protects glioma cells from ferroptosis by neutralizing DHA induced lipid peroxidation (Chen et al., 2019).

Thus, based on the existing research status, HSP70 family members may enhance cell resistance to ferroptosis by promoting GPX4 expression and its antioxidant activity, inhibiting the production of lipid ROS.

### HSP40 (DNAJ) and Ferroptosis

In all known protein homeostasis processes, HSP70 exerts chaperone activity assisted by two types of well-defined and essential chaperones: HSP40 family and nucleotide exchange factors (NEFs) (Hartl and Hayer-Hartl, 2009). Therefore, to some extent, HSP40 and HSP70 functions are inseparable. In fact, HSP40 protein determines the activity of HSP70 by stabilizing the interaction with substrate proteins. Functionally conserved HSP40 has a J-domain that interacts with HSP70 and then referred to as a J-domain-containing protein. This domain is characterized by its conserved histidine, proline and aspartate residues (Qiu et al., 2006). The binding of J domain to HSP70 enhances the ATPase activity of HSP70, thereby regulating protein folding, translation and translocation.

Based on the unique structure and precise function of HSP40, it has been shown to be highly relevant to the development of cancer. Interestingly, HSP40 family proteins have dual properties and play different roles in anticancer and cancer promotion. For example, studies on lung cancer specimens have shown that HSP40 is highly expressed in cancerous lung tissues, detection of HSP40 level in serum of tumor patients with anti-HSP antibodies can be used for tumor diagnosis (Oka et al., 2001). On the contrary, DNAJB4 (also known as HLJ1) is a tumor suppressor that can inhibit the proliferation and invasion of lung cancer cells. High DNAJB4 levels can slow down lung cancer cell cycle progression through the STAT1/P21 pathway (Wang et al., 2005; Zhang et al., 2011).

At present, there are few studies on the relationship between HSP40 family and ferroptosis. It is of pioneering significance to explore the regulation and occurrence of HSP40 family members and ferroptosis. Studies have confirmed that in esophageal squamous adenocarcinoma, overexpression of DNAJB6 enhances the degradation of GSH, down regulates GPX4, enhances lipid peroxidation and promotes ferroptosis. This confirmed the adverse role of HSP40 family members in regulating ferroptosis (Jiang et al., 2020).

Similarly, based on previous studies, we predict that, like DNAJB6, other HSP40 family members may promote the occurrence of ferroptosis by inhibiting GSH/GPX4 and enhancing lipid peroxidation. So, it may be of great significance to continue to explore the relationship between HSP40 family members and ferroptosis.

### Small Heat Shock Protein and Ferroptosis

sHSP is usually defined as an ATP independent HSP with a subunit molecular weight of 12–43 kDa. Members of the sHSP family possess a homologous core domain, a highly conserved α-crystalline domain (ACD) consisting of 80–100 amino acids (Kriehuber et al., 2010). In the sHSP family, HO-1(HSP32), HSPB1 (HSP27), HSPB4 (αA crystal protein) and HSPB5 (αB crystal protein) have been studied and reported the most. sHSP is usually present in oligomeric complexes involving one or more family members and thus may provide cells with molecular chaperone-specific diversity. This is because the most striking feature of sHSP is its oligomerization, in which sHSP adjusts its quaternary structure through the assembly of monomers or dimers (Benesch et al., 2008). They can interact with themselves or other sHSPs to form homo or hetero oligomers containing up to 50 subunits (Garrido et al., 2012). This dynamic oligomerization process can cause rapid subunit exchange. The assembly of different substructures affects the exposure of hydrophobic surfaces, thus providing a molecular mechanism to regulate their binding activity.

Ferroptosis is a process dependent on both iron and ROS. After oxidative stress, cells respond through the inherent adaptive defense mechanism to restore healthy cell redox homeostasis. One mechanism involves the haem oxygenase enzyme system, especially its inducible isomer HO-1, a member of sHSP family. This is a kind of cell protective, anti-inflammatory and antioxidant enzyme. HO-1 expression was up-regulated in cancer cells when ferroptosis occurs (Kwon et al., 2015). For example, in human renal tubular epithelial cells, HO-1 has an important anti-ferroptosis effect. Its mechanism may be that HO-1 protects AKI from ferroptosis by promoting GSH depletion (Zhang et al., 2009; Adedoyin et al., 2018). In addition, in the pathogenesis of adriamycin induced myocardial toxicity and ischemia-reperfusion mediated ferroptosis in cardiomyopathy, Nrf2/HO-1 axis promotes the release of free iron, and excessive free iron accumulates in mitochondria, resulting in lipid peroxidation on mitochondrial membrane, mitochondrial dysfunction, and then aggravate ferroptosis (Fang et al., 2019).

HSPB1, a member of sHSP, also plays an integral role in ferroptosis. Previously, HSPB1 was considered to be a negative regulator of iron accumulation and uptake in fibroblasts or cardiac cells (Chen et al., 2006; Zhang et al., 2010). Sun et al. found that HSPB1 overexpression also inhibited erastin-induced iron uptake, while down regulating HSPB1 expression increased erastin-induced iron uptake in cancer cells (Sun et al., 2015). Therefore, when cancer cells contain abnormally elevated iron and HSPB1, if HSPB1 pathway is down regulated, cells may be prone to ferroptosis (Oesterreich et al., 1993; Torti and Torti, 2013). Inhibition of HSF1-HSPB1-PRKC pathway promotes ferroptosis in cancer cells induced by erastin (Sun et al., 2015). Besides, proteomic analysis of animal models of depression found that, the levels of alpha crystallin B (Cryab) and brain-derived neurotrophic factor (BDNF) of sHSP family members decreased, can explain the loss of some neurons during ferroptosis (Cao et al., 2021). However, the mechanism involved is unclear.

Based on the relationship and discovery of existing sHSP family members in ferroptosis, it can regulate ferroptosis through iron metabolism and GSH/GPX4 pathway. This regulation may be beneficial, such as HO-1 and HSPB1; it may also be harmful, such as Nrf2/HO-1 pathway. Therefore, in-depth study of their relationship may have some enlightenment for the research progress of ferroptosis.

### Ubiquitin and Ferroptosis

Ubiquitin is a small protein with a molecular weight of 8.5 kDa and 76 amino acids. It is highly conformed in eukaryotes and named by its widespread expression in cells. There are eight amino acid sites of ubiquitin itself (Met1, Lys6, Lys11, Lys27, Lys29, Lys33, Lys48, Lys63), which can be modified by other ubiquitin molecules to form eight poly polymerized ubiquitin chains. Ubiquitin modification system is one of the most common post-translational modification (PTM) systems. It serves as a label or signal to determine the fate and/or function of its labeled substrate protein, and has complex and important physiological functions.

The process of ubiquitin modification of target protein depends on the cascade reaction catalyzed by three kinds of ubiquitin enzymes, namely, ubiquitin activase (E1) binding enzyme (E2) and ligase (E3). Firstly, E1 mediates ATP-dependent activation of ubiquitin to initiate a reaction in which E1 and the 76th amino acid glycine carboxyl group of ubiquitin converted to thiocyanates. Secondly, E2 enzyme acts on the chemical bond for thioesterification, coupling ubiquitin to cysteine residues in E2. Finally, ubiquitin attached to the lysine residue of the target protein by E3. Among them, the specificity of E3 enzyme determines the specificity of ubiquitinated substrate (Hershko and Ciechanover, 1998; Pickart and Eddins, 2004). Single ubiquitin protein can continue to link ubiquitin protein to form polymerized ubiquitin chain. The specificity of ubiquitin chain type determined by E3 enzymes and some specific E2 enzymes (Stewart et al., 2016; Swatek and Komander, 2016).

Unlike other HSPs, ubiquitin exhibits certain uniqueness in functional roles, such as protein degradation pathways (Dikic et al., 2006). As a unique member of the HSP family, ubiquitin closely related to ferroptosis. For example, ubiquitin specific protease 35(USP35) belongs to a family of de-ubiquitinase related to cell proliferation and mitosis. In lung cancer cells, USP35 interacts directly with FPN1 to maintain its protein stability and prevent iron overload and ferroptosis (Tang Z. et al., 2021). Down regulation of USP35 promotes ferroptosis and inhibits cell growth, colony formation and tumor progression. Therefore, it is a promising therapeutic target for lung cancer. 6-Gingerol improves autophagy-dependent-ferroptosis by inhibiting the expression of USP14 and increasing the contents of ROS and Fe^2+^, which verified the protective effect of USP14 on ferroptosis (Tsai et al., 2020). In addition, overexpression of USP22 can reduce ferroptosis, accompanied by an increase in GSH and a decrease in ROS production, lipid peroxidation and iron accumulation (Ma et al., 2020). In I/R treated rat hearts, USP7, p53 and TfR1 formed a unique pathway of USP7/p53/TfR1. Inhibition of USP7 can inhibit the ubiquitination process and then activate p53, resulting in the down-regulation of TfR1, accompanied by the reduction of ferroptosis and myocardial injury (Tang L.-J. et al., 2021).

In addition to the above, different members of the E3 ligase family also play important roles in regulating ferroptosis. TRIM26 is an E3 ubiquitin ligase, which plays a role as a tumor suppressor in hepatocellular carcinoma. TRIM26 can inhibit liver fibrosis by mediating the ubiquitination of SLC7A11 and promoting the ferroptosis of hepatic stellate cells (HSCs), which can be used as a new therapeutic strategy for liver fibrosis (Zhu et al., 2021). Similarly, E3 ubiquitin ligase S-phase kinase-associated protein 2(SKP2) also plays a role in ferroptosis: YAP (Yes-associated protein 1) is the only analogue of TAZ (a regulator of the Hippo pathway, which regulates ferroptosis in renal and ovarian cancer cells), which promotes the production of ROS by regulating the SKP2 thus aggravates ferroptosis. It provides a new treatment for YAP driven tumors (Yang et al., 2021).Besides, the ubiquitin-E3 ligase TRIM21 interacts with and ubiquitinizes p62, negatively regulating the antioxidant pathway of p62-keap1-Nrf2, thereby exacerbating ferroptosis (Hou et al., 2021). Knockout of TRIM21 protects cardiomyocytes from doxorubicin-induced ferroptosis. However, from the perspective of mechanism, it should be noted that, as a ubiquitin-E3 ligase, TRIM21 can promote proteasome degradation of various proteins, and may play an anti-ferroptosis role through regulating other pathways, one of which may be regulated by the immune system (Hou et al., 2021). Similarly, as a member of the E3 ligase family, neuronal precursor cell expressed developmentally downregulated 4(Nedd4) is an important member of this family. Down-regulation Nedd4 saves erastin-induced elimination of the voltage-dependent anionic channel VDAC2/3 protein, increases the resistance of melanoma cells to erastin (Yang et al., 2020).

To sum up, ubiquitin family proteins closely related to the mechanism of ferroptosis, which may involve many cytokines or mechanisms, including GSH/GPX4 antioxidant pathway and iron metabolism, etc. The continued study of ubiquitin system has prospective significance and broad prospects for mastering the regulatory mechanism of ferroptosis.

### Perspectives

**FIGURE 1:**
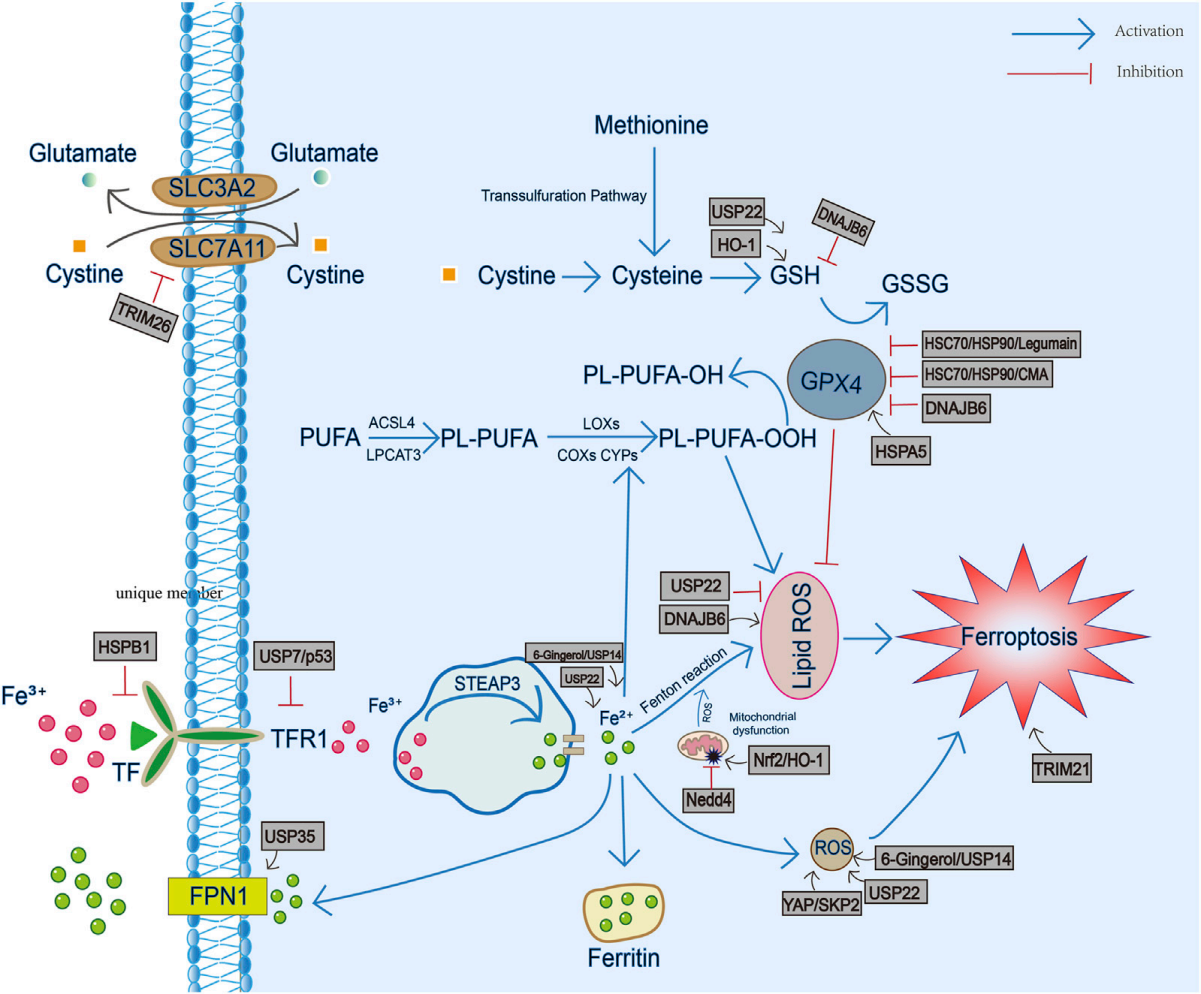
The involvement of heat shock proteins in iron metabolism, GSH/GPX4 antioxidant pathway and production of reactive oxygen species and lipid peroxidation during ferroptosis. TF, transferrin; TFR1, transferrin receptor 1; FPN1, ferroportin1; PL-PUFA, phospholipid-bound polyunsaturated fatty acids; STEAP3, six-transmembrane epithelial antigen of prostate 3; ROS, reactive oxygen species; GSH, glutathione; GSSG, oxidized glutathione; GPX4, Glutathione peroxidase 4.

In conclusion, after the above review, we summarized and predicted the mechanism of different HSP family members in ferroptosis (Figure 1). It seems that each HSP family has members either promote ferroptosis (such as HSP90 family, HSP40 family, partial members of sHSP family and partial ubiquitin ligases) or inhibit ferroptosis (such as HSP70 family, partial members of sHSP family and some ubiquitin ligases) by targeting the GSH/GPX4 antioxidant pathway. In addition to GSH/GPX4 axis, other antioxidant pathways, such as FSP1/CoQ10 axis and GCH1/BH4 axis, also have great exploration potential. Therefore, future studies can target these antioxidant pathways to explore the association between HSP and ferroptosis.

## Summary and Prospect

The research on fine regulation mechanism related to ferroptosis is increasing rapidly. However, different HSP family members seem to regulate ferroptosis differently in different diseases/organ microenvironments. In this review, we summarized the effects of HSP on ferroptosis in existing studies. They may participate in the regulation of ferroptosis through iron metabolism, GSH/GPX4 axis, production of ROS and lipid peroxidation. Future research needs to determine the pathophysiological effects of HSP family in ferroptosis, especially in tumorigenesis and neurodegenerative diseases, so as to provide new ideas and strategies for defining the new mechanism of ferroptosis.
